# Dr. Wilhelm Ebstein

**Published:** 1913-01

**Authors:** 


Geheimrat Dr. Wilhelm Ebstein, Prof. of Internal Medicine at the University of Gottingen, 1877-96, died Oct. 22, aged 76. He was especially known for his work on obesity.

